# Occupancy data improves parameter precision in spatial capture–recapture models

**DOI:** 10.1002/ece3.9250

**Published:** 2022-08-26

**Authors:** José Jiménez, Francisco Díaz‐Ruiz, Pedro Monterroso, Jorge Tobajas, Pablo Ferreras

**Affiliations:** ^1^ Instituto de Investigación en Recursos Cinegéticos (IREC, CSIC‐UCLM‐JCCM) Ciudad Real Spain; ^2^ Departamento de Biología Animal, Facultad de Ciencias Universidad de Málaga Málaga Spain; ^3^ CIBIO, Centro de Investigacão em Biodiversidade e Recursos Genéticos, InBIO Laboratório Associado Universidade do Porto Vairão Portugal; ^4^ BIOPOLIS Program in Genomics, Biodiversity and Land Planning CIBIO Vairão Portugal; ^5^ Departamento de Botánica, Ecología y Fisiología Vegetal Universidad de Córdoba Córdoba Spain

**Keywords:** camera traps, integrated spatial capture–recapture model, stone marten, telemetry, terrestrial passive integrated transponder, unmarked species

## Abstract

Population size is one of the basic demographic parameters for species management and conservation. Among different estimation methods, spatially explicit capture–recapture (SCR) models allow the estimation of population density in a framework that has been greatly developed in recent years. The use of automated detection devices, such as camera traps, has impressively extended SCR studies for individually identifiable species. However, its application to unmarked/partially marked species remains challenging, and no specific method has been widely used. We fitted an SCR‐integrated model (SCR‐IM) to stone marten *Martes foina* data, a species for which only some individuals are individually recognizable by natural marks, and estimate population size based on integration of three submodels: (1) individual capture histories from live capture and transponder tagging; (2) detection/nondetection or “occupancy” data using camera traps in a bigger area to extend the geographic scope of capture–recapture data; and (3) telemetry data from a set of tagged individuals. We estimated a stone marten density of 0.352 (SD: 0.081) individuals/km^2^. We simulated four dilution scenarios of occupancy data to study the variation in the coefficient of variation in population size estimates. We also used simulations with similar characteristics as the stone marten case study, comparing the accuracy and precision obtained from SCR‐IM and SCR, to understand how submodels' integration affects the posterior distributions of estimated parameters. Based on our simulations, we found that population size estimates using SCR‐IM are more accurate and precise. In our stone marten case study, the SCR‐IM density estimation increased the precision by 37% when compared to the standard SCR model as regards to the coefficient of variation. This model has high potential to be used for species in which individual recognition by natural markings is not possible, therefore limiting the need to rely on invasive sampling procedures.

## Graphical abstract

We fitted a spatial capture–recapture integrated model (SCR‐IM) to stone marten (*Martes foina*) data, and estimate population size based on integration of three submodels: (1) individual capture histories from live capture and transponder tagging; (2) detection/nondetection or “occupancy” data using camera traps in a bigger area to extend the geographic scope of capture–recapture data; and (3) telemetry data from a set of tagged individuals. The SCR‐IM density estimation improved the precision by 37% when compared to the standard SCR model.

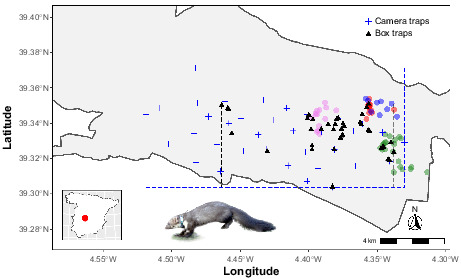

## 1 INTRODUCTION

Population size is one of the key demographic parameters for species management and conservation. While many methods for estimating wildlife population size have been proposed, capture–recapture (CR) models have long been considered the gold standard (Otis et al., 1978; Pollock, 1976). However, under the conventional CR formulation, the area to which the population size estimate should be allocated is unknown and arbitrarily defined because of ambiguity in the criteria for delimiting the effective sampling area to which captures should be referenced (Efford, 2004). Recently, a set of spatial capture–recapture (SCR)‐based models have been developed that explicitly incorporate spatial information into abundance calculations, and thus allow proper density estimates (Efford, 2004; Royle et al., 2014). These include spatial mark‐resight (SMR) methods, which were purposely developed to deal with partially marked populations (both natural and human‐derived marks) (Sollmann, Gardner, Chandler, et al., 2013; Sollmann, Gardner, Parsons, et al., 2013). Alongside these analytical developments, the widespread use of camera traps for wildlife research, combined with the use of SCR models, has allowed density estimation for species whose individuals are individually identifiable. Likewise, genetic analyses have generalized the use of SCR with genotype‐based identification (e.g., Gardner et al., 2010; Kéry et al., 2011).

The convenience of using all available data to improve inferences, avoiding discarding, for example, imperfect identifications, has motivated the development of specific models to deal with these problems. Spatial partial identity models (SPIMs) (Augustine et al., 2018, 2019, 2020) extended the domain of SCR to populations or observation systems that do not always allow for individual identity to be determined with certainty, while the random thinning SCR model was developed as evolutions of the standard SCR models to deal with incompletely identified data (Jiménez et al., 2021). The latter model allows incorporating detection events with unknown marking status, which are discarded in standard SCR and SMR approaches. To avoid violating the assumption from SMR that marked and unmarked populations have the same encounter probabilities, Whittington et al. (2017) developed the generalized spatial mark‐resight (gen‐SMR) model, that involved the integration of data obtained from different sampling methods, using live‐trapping capture histories and camera‐trapping resighting histories. Although this integration was primarily aimed at overcoming the assumption of equal encounter probabilities, its data integration also improved the precision of the estimated parameters by accommodating additional sources of data (Schaub & Kéry, 2012; Whittington et al., 2017). The generalized spatial mark‐resight model with incomplete identification (gen‐SMR‐ID: Jiménez, Chandler, et al., 2019) was developed as a natural evolution of the gen‐SMR model that allows integrating live‐trapping, photo‐trapping, and telemetry data in a single unified modeling approach. Both gen‐SMR and gen‐SMR‐ID approaches are paradigmatic of the improvement in population estimates obtained by data integration under the SCR (or SMR) modeling framework. However, the origins of data integration in SCR and SMR models regard to the improvement of the half‐normal scale parameter (σ) that describes how detectability decreases with distance from the center of activity of each individual. The integration of telemetry data was a natural way of improving σ estimates, in both SCR (Jiménez et al., 2017; Linden et al., 2018) and SMR (Jiménez, Chandler, et al., 2019; Royle et al., 2014; Sollmann, Gardner, Parsons, et al., 2013), and indirectly change the estimates of the baseline detection rate ($\lambda_{0}$) since both parameters are related in the structure of the detection submodel (Dey et al., 2019; Efford & Mowat, 2014). In the field of dynamic models, integrated population models (IPMs) are a whole family of demographic models that include multiple sources of data to estimate different demographic parameters (Schaub & Kéry, 2022). IPMs represent a remarkable methodological advancement (Schaub & Kéry, 2012), and have proved useful in improving our ecological understanding of population processes, and in improving management decisions (e.g., Bled et al., 2017; Rushing et al., 2017). IPMs in spatial capture–recapture combine, for example, SCR and distance sampling data (Chandler et al., 2018), and SCR and dead recovery data (Dupont et al., 2021). IPMs allow combining “cheap and risky” data (e.g. detection/non‐detection) with more “expensive and reliable” data sets (e.g. capture–recapture), reconciling potential spatial and temporal misalignments with a unified modeling structure that explicitly describes each component (i.e., dataset). While usually less informative, “cheap” data are generally easier to collect and allow for larger spatiotemporal sampling scales. Conversely, “expensive” is more informative but tends to be logistically challenging and/or expensive to obtain, which typically constrains the sampling approach to a smaller spatial or temporal scale. When combined, these datasets may mitigate each other's weaknesses, allowing for an increase in the scope of inference (Kéry & Royle, 2021, chapter 10).

Despite this highly prolific decade in model development, an “integrated model” (IM) specifically aimed at improving the estimation of the state parameters, that is abundance, was first introduced in an SCR model by Kéry and Royle (2021). The model described by these authors relies on a joint likelihood for multiple data sets describing a shared state process but different observation processes. Two different spatial capture–recapture integrated models were presented, and tested with simulated data: (1) the integrated SCR‐Counts model, which integrates capture histories and count data without identification, and (2) the integrated SCR‐Occ model, which integrates of SCR and “occupancy” (detection/nondetection) data. The SCR‐Counts model was used by Ferreras et al. (2021) to estimate European wildcat *Felis silvestris* population size in Cabañeros National Park (Spain), demonstrating its applicability in extremely low densities. To the best of our knowledge, the SCR‐Occ was only tested on simulated data, and therefore requires an implementation with real‐life datasets (Kéry & Royle, 2021). Here, we use the SCR‐Occ model, and an extended version of it that includes a telemetry submodel, fit them to a real stone marten *Martes foina* dataset, and quantify their benefits in improving parameter precision when compared to the standard SCR model.

Stone marten density is relatively understudied in Mediterranean habitats, and more so using SCR models, probably by the difficulties in individual identification (but see Jiménez et al., 2017; Jiménez, Nuñez‐Arjona, et al., 2019). Stone martens have very subtle individual markings, which make them difficult to individually identify. As described for the American marten *Martes americana* (Sirén et al., 2016), stone marten's individual throat patch pattern is hardly visible in ordinary camera‐trapping images. Alternative sampling procedures allowing the use of SCR models are tagging‐based, and included live capture and tagging with collars, ear tags, or passive integrated transponders (henceforth transponders). However, according to our preliminary tests with this species, the durability of collars and ear tags in stone marten is minimal and the loss of marks could bias population estimates. Conversely, transponder tagging would require a large live trap sampling grid, which would entail a prohibitively expensive and invasive sampling operation. To overcome the above‐mentioned limitations, we implemented a modification of the SCR‐Occ model described by Kéry and Royle (2021) by adding a telemetry submodel to it (Royle et al., 2014, p. 516). Thus, the goals of this paper were: (i) making a proof of concept of the SCR‐Occ model with a real data; (ii) using simulations with similar values to our real dataset and outputs, calculate bias and precision in all models estimates; (iii) quantify changes in precision of the parameters estimates when compared to standard SCR models in our case study; (iv) evaluate the feasibility of using this approach to species with very subtle individual markings; and (v) generating new estimates of density for a widespread and ecologically important species, for which estimates of such vital rates are typically absent.

## 2 MATERIALS AND METHODS

### 2.1 Study area and species

The study was carried out in Cabañeros National Park (henceforth Cabañeros) located in central Spain (39°24′N; 4°29′W). With altitudes between 560 and 1448 m, the park features 40,000 ha of well‐preserved Mediterranean ecosystems. Climate is Mediterranean, with moderately rainy springs and autumns (annual rainfall 450–750 mm) and hot dry summers and mild winters. Vegetation is dominated by scrublands of rockrose *Cistus* spp, *Phillyrea angustifolia*, strawberry trees, and *Erica* spp., and the tree layer is dominated by holm (*Quercus rotundifolia*), gall (*Quercus faginea*), and cork oaks (*Quercus suber*). The central area of Cabañeros (known as “raña”) is a savanna‐like open tree layer with scattered holm, gall, and cork oaks.

The stone marten has a wide distribution, extending over almost the entire mainland Europe and some parts of Asia (Abramov et al., 2016). Its ecological adaptability deems it present in a wide variety of habitats (Abramov et al., 2016; Virgós et al., 2012). It has a remarkable ecological role in the dispersal of multiple fleshy fruited plants (such as strawberry tree, *Arbutus unedo*) in Mediterranean ecosystems (Burgos et al., 2022; Herrera, 1989; Virgós et al., 2010), where stone martens play a potential role as ecosystem engineer. Their ecological importance coupled with its drastic decline where the Iberian lynx *Lynx pardinus* has settled after its reintroduction in the Iberian Peninsula (Jiménez, Nuñez‐Arjona, et al., 2019), justify the need to accurately quantify stone martens' population sizes, as a key step toward a deeper understanding of the seed dispersal processes in which it is involved, and the consequences of intraguild relationships in those processes. Given their wide geographic range, understanding their ecological role has relevance across vast regions and ecosystems.

Red deer (*Cervus elaphus*) and wild boar (*Sus scrofa*) were at high densities at the study area, although no population estimates were available. The mammalian carnivore community is dominated by red fox, which is the most abundant species, with 0.947 (SD: 0.156) individuals/km^2^ (Jiménez, 2021). Other species present are the common genet (*Genetta genetta*), the European badger (*Meles meles*), the Egyptian mongoose (*Herpestes ichneumon*), the otter (*Lutra lutra*), the least weasel (*Mustela nivalis*), the polecat (*Mustela putorius*), and the wildcat (Ferreras et al., 2016, 2017).

### 2.2 Live capture and tagging

**FIGURE 1:**
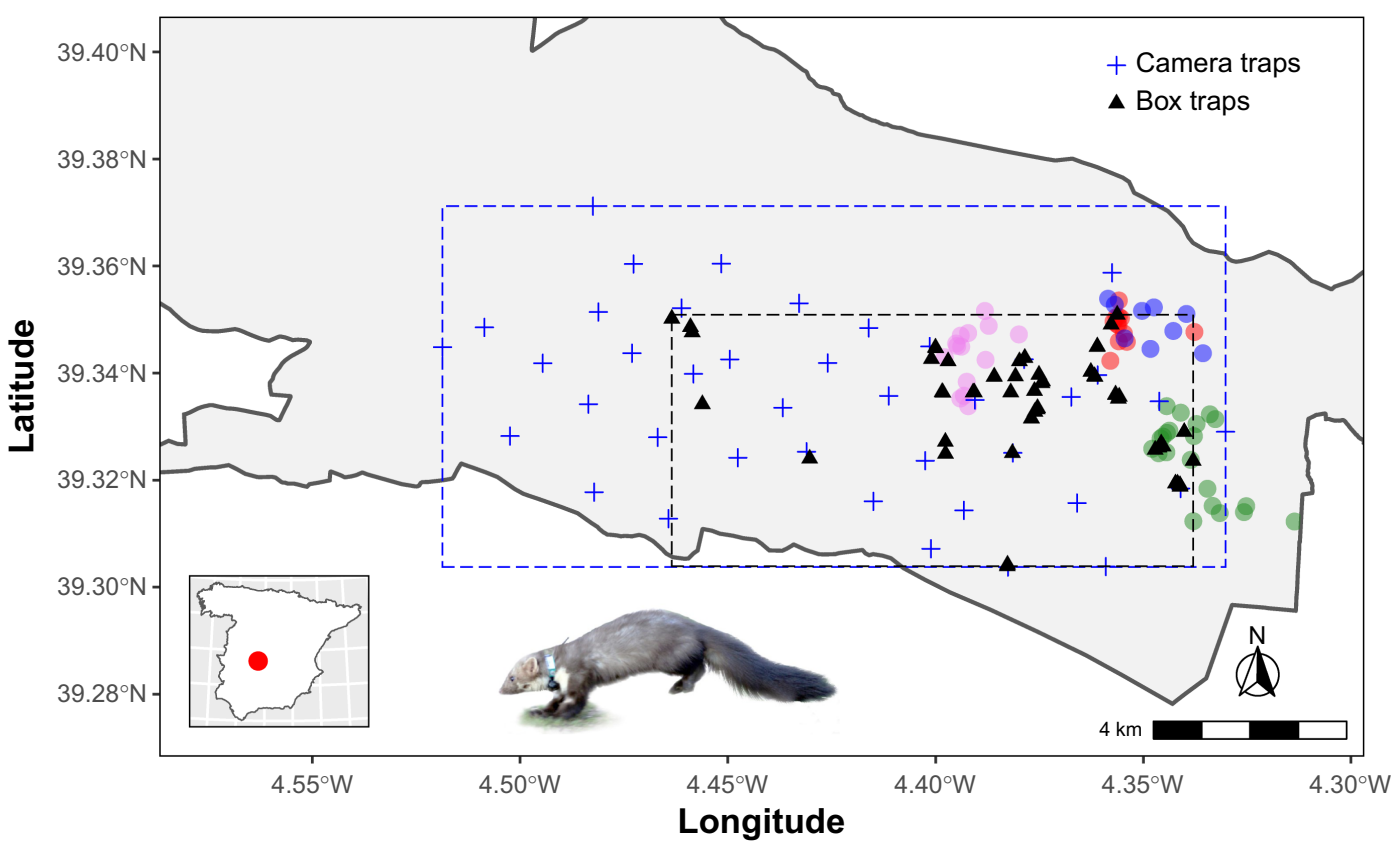
Spatial arrangement of camera traps (blue crosses) and live traps (black triangles) and their respective geographical envelopes (blue and black dashed rectangles, respectively); thinned GPS telemetry positions (violet, red, blue, and green) for four stone martens at the South of Cabañeros National Park (gray shadowed area). Bottom‐left, location of the study area in Spain.

Stone martens were captured using box traps, including Tomahawk (Model 208, Tomahawk Live Trap, WI, USA) and two models (Jauteco and Alvega) of wire mesh traps from local dealers, with the required animal care permits for live captures (approved code PR‐2013‐05‐04 from the Ethical Committee on Animal Testing of Castilla‐La Mancha University). Traps were baited either with dead bait (chicken) or with live red‐legged partridges (*Alectoris rufa*) or house pigeons (*Columba* sp.). Live baits were placed in an independent chamber inaccessible to captured carnivores, provided with water and food ad libitum and covered with small branches to protect them from inclement weather, following EU recommendations regarding animal welfare. We deployed a total of 60 box traps (Figure 1) in two trapping campaigns between March 3th and July 4th 2014 (Figure S1). Box traps were placed within an envelope (rectangular area encompassing all the traps) of 5652 ha in locations potentially suitable for stone martens according to our knowledge of the species, at an average intertrap distance of 177 m (range 4–2499 m). Because of the nocturnal activity of stone martens (Monterroso et al., 2014), box traps were checked daily after sunrise to minimize animal stress. Captured stone martens were immobilized with a combination of medetomidine hydrochloride (Medetor, Virbac, Spain) and ketamine hydrochloride (Imalgene 1000, Merial, Spain) with average dosages of 0.07 mg/kg and 9.55 mg/kg, respectively. We used atipamezole (Antisedan, Pfizer, Spain) at a dose of 0.35 mg/kg to reverse the effects of medetomidine and accelerate recovery (Gunkel & Lafortune, 2007). All captured stone martens were tagged with a microtransponder (ID‐100A, Trovan) injected subcutaneously in the neck side for its identification in subsequent recaptures. Four stone martens were also equipped with VHF‐GPS radio collars (66 g, model TGB‐316, Telenax, Mexico). Stone martens were released where captured once fully recovered from anesthesia, always within three hours after capture. Fixes for the radio‐tagged stone martens were attempted daily through triangulation of the VHF signal and retrieved from the GPS units.

### 2.3 Camera‐trapping

We deployed 40 camera‐trapping stations (one camera per station) between January 15 and April 22, 2014 (Figure 1). Stations were in a grid distribution such that each camera, on average, was 1267 m from its nearest neighbor (range: 712–2143 m), covering a geometrical envelope of 12,174 ha. The camera‐trapping grid partially overlapped the live‐trapping grid (Figure 1), covering the same habitat types, but not colocated with live traps avoiding data dependence (Clare et al., 2017). We used two low‐glow infrared camera‐trap models, namely, ScoutGuard SG550 and SG570 (HCO Outdoor Products), with similar performing features (e.g., 1.2–1.3 s trigger speed). Cameras were secured inside metal boxes, locked with a cable lock and attached to a tree approximately 50 cm above ground. As attractant, we placed Iberian lynx urine and valerian extract in separate vials, a combination proved effective for Iberian mesocarnivores including stone martens (Ferreras et al., 2018; Monterroso et al., 2011), at a distance of 2–3 m from the camera traps. We programmed cameras to shoot a burst of three photos when triggered, with medium sensitivity and minimal delay time (0 s). Camera traps remained active between 52 and 98 days (Figure S1). Consecutive photo‐captures of stone martens in a given camera within a 30‐min interval were considered as the same event (Jiménez et al., 2017).

### 2.4 Statistical modeling

We tested if the use of dead vs. live bait in box traps changed the baseline detection rate ($\lambda_{0}$) in the SCR model (Royle et al., 2014) using a trap‐level covariate:

$$\text{logit}\left(\lambda_{0_{j}}\right)=\beta_{0}+\beta_{1}\times b$$

where $\beta_{0}$ was the intercept and $\beta_{1}$ was the bait effect (death vs. live bait) by trap, and b (bait) was a vector with two possible values: 0 (dead bait) and 1 (live bait). We used reversible jump MCMC (RJMCMC) in NIMBLE version 0.12.2 (NIMBLE Development Team, 2019) to decide whether or not to include this covariate, which is a natural application in Bayesian variable selection problems (see BUGS code, in Appendix S1).

After this preliminary model selection, we used an SCR‐IM combining three data sources: (i) capture histories with three dimensions (individual‐trap‐occasion) from live trapping in a core area, (ii) detection/nondetection data with two dimensions (trap‐occasion) from camera traps in a larger area (Kéry & Royle, 2021, p. 636); and (iii) telemetry data from VHF‐ and GPS‐tagged individuals (Sollmann, Gardner, Parsons, et al., 2013; Royle et al., 2014, p. 516).

The SCR and occupancy submodels shared the same underlying point process by which we assumed that individual activity centers i=1,2,…,N are distributed over a state space $\left(S\right)$ and that individuals are exposed to sampling by detector traps with location $x_{j}$ within S. We assumed the distribution of individuals' activity centers $s_{i}=\left(s_{i_{1}},s_{i_{2}}\right)$ to be described by a homogeneous point process such that $s_{i}\sim \text{Uniform}\left(S\right)$ that does not change during the *k* sampling occasions. The function describing the encounter rate $\lambda_{\mathrm{ij}}$ of individual i having activity center $s_{i}$, in trap j, is defined as:

$$\lambda_{\mathrm{ij}}=\lambda \left(s_{i},x_{j}\right)=\lambda_{0}\cdot \exp\left(-\frac{d_{\mathrm{ij}}^{2}}{2\sigma^{2}}\right)$$

where $d_{\mathrm{ij}}$ is the Euclidean distance between trap location $x_{j}$ and activity center of the individual $s_{i}$, and σ is the spatial scale parameter of the half‐normal detection function that describes the animal movement. This implies that the detection probability of a given individual in each trap declines monotonically with the trap's distance to its activity center.

We “quantized” (Royle et al., 2014, p. 249) individual encounter frequencies (truncating to binary observations by transforming daily counts to 0/1) to avoid violating the independence assumption because counts in camera traps are usually unrelated to the fundamental space usage process that underlies the genesis of SCR data (Royle et al., 2014, section 9.1.3), and we used a Bernoulli model in SCR and occupancy submodels of the SCR‐Occ. Thus, encounter histories for SCR data are binary such that:

$$y_{\mathrm{ijk}}\sim \text{Bernoulli}\left(p_{\mathrm{ijk}}^{\mathrm{scr}}\right)$$

where the observed data $y_{\mathrm{ijk}}$ is the realization of a Bernoulli process with probability $p_{\mathrm{ijk}}^{\mathrm{scr}}$, which is the detection probability defined by a complementary log–log link function that relates it to the detection rate $\lambda_{\mathrm{ijk}}$ as:

$$p_{\mathrm{ijk}}^{\mathrm{scr}}=1-\exp\left(-\lambda_{\mathrm{ijk}}\right)$$

Under a Bayesian approach to capture–recapture with unknown N, “data augmentation” can be used to estimate the number of unobserved individuals (Royle et al., 2014). We added to the n‐observed encounter histories a collection of M–n “all‐zero” histories, choosing an M value such that M≫N. The likelihood for the zero‐inflated true encounter frequencies is then modified by a partially latent binary indicator variable $z_{i}$ that describes the membership of individual i to the population. Under this specification, $\mathrm{Pr}\left(z_{i}=1\right)=1$ for the n observed individuals, and $z_{i}\sim \text{Bernoulli}\left(\psi \right)$ for the entire collection of M individuals. Population size can then be derived from the sum of indicators, $N=\sum z_{i}$ (realized *N*) or from the product M·ψ (expected *N*), and density can be derived by dividing population size by the surface area of the state space, $D=N/\left\|S\right\|$.

The occupancy submodel for detection/nondetection data ${\mathrm{occ}}_{\mathrm{jk}}$ is defined as:

$${\mathrm{occ}}_{\mathrm{jk}}\sim \text{Bernoulli}\left(1-\prod_{i=1}^{M}\left(1-p_{\mathrm{ijk}}^{\mathrm{occ}}\cdot z_{i}\right)\right)$$

This model states that detection occurs at camera trap *j* at occasion *k* if at least one individual in the population is detected, but the identity of the individual is unknown. $p_{\mathrm{ijk}}^{\mathrm{occ}}$ is analogous to $p_{\mathrm{ijk}}^{\mathrm{scr}}$ but with latent identity of individuals, while $\lambda_{0}$ is different between detection methods (box traps and camera traps). The SCR and occupancy submodels of the SCR‐IM share the same Poisson point process ($s_{i}$) over the state space *S* and the scale parameter σ of the detection function, following the key principle of a typical IM: “same process, different observation model” (Kéry & Royle, 2021, p. 636).

For computational efficiency, we assumed no temporal variation in the detection process, and consequently aggregated the binary encounter histories from SCR and occupancy submodels over $K_{j}$—a vector of sampling occasions of each trap—and recorded the total number of encounters out of $K_{j}$ (see BUGS codes, in Appendix S1). Therefore, in SCR, ${y_{\mathrm{scr}}}_{\mathrm{ij}}$ values are assumed as mutually independent outcomes of a binomial random variable such that:

$${y_{\mathrm{scr}}}_{\mathrm{ij}}\sim \text{Binomial}\left(K_{j},p_{\mathrm{ij}}^{\mathrm{scr}}\right)$$

And similarly, for occupancy model:

$$y_{{\mathrm{occ}}_{j}}\sim \text{Binomial}\left(K_{j},\left(1-\prod_{i=1}^{M}\left(1-p_{\mathrm{ij}}^{\mathrm{occ}}\right)\right)\right)$$

As our detection function is half‐normal function, we can relate the parameters σ and $s_{i}$ directly to those from a bivariate normal (BVN) movement model, where the mean is $s_{i}$, the variance—in both dimensions—is $\sigma^{2}$, and covariance is 0 (Sollmann, Gardner, Parsons, et al., 2013). If $R_{i}$ locations $I_{i}$ (of coordinates $l_{\mathrm{irx}}$ and $l_{\mathrm{iry}}$) of individual i can be represented as:

$$I_{i}\sim \mathrm{BVN}\left(s_{i},\sigma^{2}I\right)$$

and we can estimate σ and $s_{i}$ from telemetry data of the m tagged individuals' R locations, using:

$$\left[\sigma |I,s\right]\propto \left[\prod_{i=1}^{m}\prod_{r=1}^{R_{i}}\frac{1}{2\pi \sigma^{2}}\exp\left(-\frac{1}{2}\left[\frac{{\left(l_{\mathrm{irx}}-s_{\mathrm{ix}}\right)}^{2}}{\sigma^{2}}+\frac{{\left(l_{\mathrm{iry}}-s_{\mathrm{iy}}\right)}^{2}}{\sigma^{2}}\right]\right)\right]\times \left[\sigma \right]$$

$$\left[s_{i}|I,\sigma \right]\propto \left[\prod_{r=1}^{R_{i}}\frac{1}{2\pi \sigma^{2}}\exp\left(-\frac{1}{2}\left[\frac{{\left(l_{\mathrm{irx}}-s_{\mathrm{ix}}\right)}^{2}}{\sigma^{2}}+\frac{{\left(l_{\mathrm{iry}}-s_{\mathrm{iy}}\right)}^{2}}{\sigma^{2}}\right]\right)\right]\times \left[s_{i}\right]$$

Information on the locations of tagged individuals collected by telemetry tags were also added to our model to further improve estimates of SCR‐Occ parameters. We assumed that telemetry data regards to thinned outcomes of a movement model with a stationary bivariate normal utilization distribution (Royle et al., 2013). Temporal dependence among telemetry locations may cause underestimation of the variance of σ and population density (Murphy et al., 2019; Sollmann, Gardner, Parsons, et al., 2013). Therefore, it was minimized by thinning to one randomly selected location per survey day for each tagged stone marten.

As real density, sigma and baseline detection rate are not known, we assess model performance by the precision of parameters estimates, using the coefficient of variation (CV, defined as the posterior standard deviation divided by the posterior mean) for the four models: SCR‐occupancy‐telemetry (SCR‐Occ‐Tel), SCR‐occupancy (SCR‐Occ), SCR‐telemetry (SCR‐Tel), and conventional SCR. We “degraded” the capture histories SCR data to detection/no‐detection and fitted a site‐occupancy model to these data for comparison with site‐occupancy outputs from camera trapping data. We then quantified the overlap of posterior parameters distribution from both occupancy under the assumption that occupancy is related to abundance (Efford & Dawson, 2012; Gaston & Blackburn, 2003), but see Steenweg et al. (2018). Minimal or not overlap of posterior parameters distribution would indicate different ecological processes. Telemetry data were analyzed in the same way. We also “diluted” our occupancy data by randomly removing 20%, 40%, 60%, and 80% of the total detections, using 77, 58, 38, and 19 events, respectively, to evaluate the relationship between occupancy sample size and CV reduction in population size estimates. For each category, we simulated 100 different datasets, using a binomial distribution for data dilution and fixing the number of detections.

To test the performance of each model, we carried out a simulation study to compare the SCR, SCR‐Occ, SCR‐Tel and SCR‐Occ‐Tel using the same camera traps and box traps deployment coordinates used in the stone marten proof‐of‐concept approach. We based our simulation scenarios on the following: (i) similar density and sigma values as estimated from our proof‐of‐concept model (see results section below); (ii) combined baseline detection rate and number of sampling occasions to get 21 captures in box traps; (iii) fixed 4 telemetry‐tagged individuals (20 locations each); (iv) similar number of camera trapping detections as obtained in our empirical data set (mean 97; range: 72–117); and (v) same priors and data augmentation parameters as in our proof‐of‐concept model. Using these specifications, we generated activity center distributions over 100 simulated scenarios, to which we fitted all four models (see Appendix S1). We then compared the results through relative bias and root‐mean‐square error (RMSE), using the R package SimDesign (Chalmers & Adkins, 2020).

We used the goodness‐of‐fit (GoF) of the SCR‐IM model to diagnose severe violations to model assumptions, and hence reduce the risk of drawing erroneous inference (Pradel et al., 2005), by computing the Bayesian *p*‐value (BPV) based on measuring the systematic dissimilarity between observed data and posterior predictive distribution of the data (Gelman et al., 1996). Thus, the computation of BPVs requires specifying a discrepancy measure by choosing the aspects of the model to be checked. Following Royle et al. (2014) (p. 232), we selected three statistics to evaluate the observation model: (1) Individual per detector frequencies; (2) Individual encounter frequencies, and (3) Trap encounter frequencies. We fit all models in NIMBLE version 0.12.2 (NIMBLE Development Team, 2019) (see BUGS codes, in Appendix S1) in R version 4.1.3 (R Core Team, 2022). We ran three chains of 50,000 iterations after an initial 10,000 as a burn‐in for all models. We ran three chains of 6000 iterations after an initial 1000 as burn‐in for all simulations. We assessed model convergence by visually examining trace plots and using estimates of effective sample size and split‐chain Rhat, which can be used to better diagnose convergence failure of MCMC chains (Vehtari et al., 2020). All modeling outputs are presented as posterior mode (SD; standard deviation), unless clearly stated otherwise.

## 3 RESULTS

### 3.1 Sampling

We captured 14 stone martens 21 times (nine individuals, once; four individuals, twice, and one individual, four times) in 1034 sampling days using box traps (03/03/2014–04/07/2014) (Figure S1). We recorded 4 spatial recaptures (captures of the same individual at different traps). In camera traps, we recorded 101 independent detections out of 3670 camera‐sampling days (Figure S2), and retained 96 camera‐trap detections after the quantizing procedure. We collared four stone marten individuals from the box‐trapping cohort. After data thinning, telemetry produced 12, 9, 21, and 14 usable locations (Figure 1).

### 3.2 Previous test and SCR models

**FIGURE 2:**
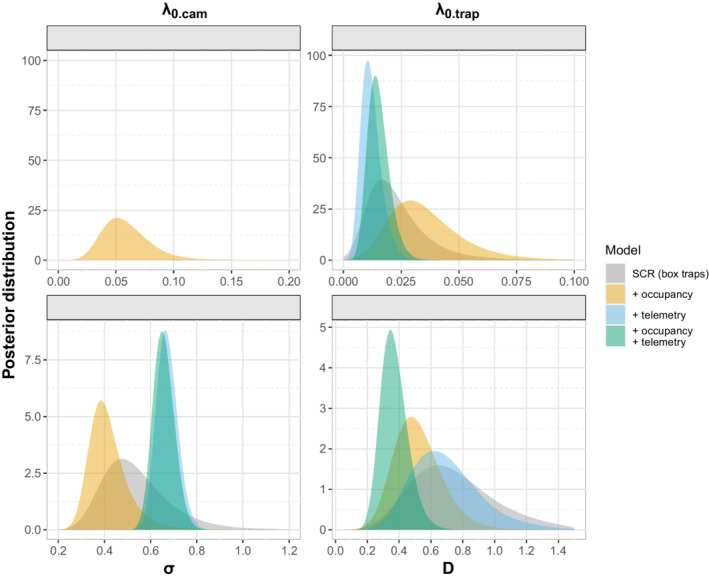
Density probabilities for models' parameters: baseline detection rate ($\lambda_{0.\mathrm{cam}}$) in camera traps; baseline detection rate ($\lambda_{0.\text{trap}}$) in box traps; scale parameter for half‐normal detection function (σ), and density (D). In gray, SCR model alone, using data histories from box traps (SCR); in gold, SCR and occupancy data (SCR‐Occ); in blue, SCR and telemetry data (SCR‐Tel); and in green, full integration of SCR, occupancy, and telemetry data (SCR‐Occ‐Tel).

The RJMCMC revealed a very low probability (0.021) of including lure as a model covariate, and therefore we decided to use the null SCR model in subsequent analyses. Posterior occupancy estimates from box traps ($\hat{\psi }=0.78;\mathrm{SD}:0.14$) and camera traps $\left(\hat{\psi }=0.66;\;\text{SD:}\;0.08\right)$ distribution originated from the camera traps. The posterior estimates from each submodel (SCR, occupancy and telemetry) largely overlapped for all parameters (Figure 2).

The SCR‐Occ‐Tel, with a CV = 0.22 (Table 1) led to the highest reduction in the CV by 37% and 76% in density and sigma estimates, respectively, compared with the standard SCR model. The second‐best performing model regarding CV reduction was the integrated SCR‐Occ, which accounted for a 19% and 26% CV reduction for the same parameters. The SCR‐Tel still provided a 13% and 76% CV reduction (Table 1 and Figure 2). The CV for σ estimates was lower in models that integrated telemetry data (Table 1). This improvement was propagated to their baseline detection rate ($\lambda_{0.\text{trap}}$) estimate (37% and 41% of CV reduction for the SCR‐Occ‐Tel and SCR‐Tel models, respectively) and only to a small degree (13% for the SCR‐Tel model) to the density estimate (Figure 2 and Table 1). Change due to occupancy submodel was similar for all parameters (ca. 20%), and the CV reduction in $\lambda_{0.\mathrm{cam}}$ is similar to $\lambda_{0.\text{trap}}$ from the SCR‐Occ to the SCR‐Occ‐Tel model. We found a pattern of lower density estimates with occupancy data integration, and higher sigma estimates when telemetry data are included.

**TABLE 1:** Parameter estimates from stone marten using spatial capture–recapture‐integrated model with detection/nondetection and telemetry data (SCR‐Occ‐Tel); spatial capture–recapture and detection/nondetection data (SCR‐occupancy); spatial capture–recapture and telemetry data (SCR telemetry) and spatial capture–recapture (SCR) data alone. Population density ($\hat{D}$; individuals/km^2^); scale parameter of the detection function ($\hat{\sigma }$; km); baseline detection rate for box traps (${\hat{\lambda }}_{0.\text{trap}}$) and camera traps (${\hat{\lambda }}_{0.\mathrm{cam}}$) are compared.

Model	Param.	Mean	Mode	SD	95% CRI		CV	Red. CV (%)
					q2.5	q97.5		
SCR‐occupancy‐telemetry)	$\hat{D}$	0.370	0.352	0.081	0.236	0.554	0.219	37.3
	$\hat{\sigma }$	0.657	0.652	0.043	0.579	0.748	0.065	76.2
	$\lambda_{0.\text{trap}}$	0.015	0.014	0.004	0.008	0.025	0.267	52.8
	$\lambda_{0.\mathrm{cam}}$	0.029	0.027	0.007	0.018	0.044	0.241	37.0
SCR‐occupancy	$\hat{D}$	0.520	0.472	0.147	0.284	0.855	0.283	19.1
	$\hat{\sigma }$	0.419	0.383	0.085	0.300	0.635	0.203	26.3
	$\lambda_{0.\text{trap}}$	0.036	0.028	0.016	0.014	0.075	0.444	21.4
	$\lambda_{0.\mathrm{cam}}$	0.059	0.048	0.021	0.029	0.109	0.356	—
SCR‐telemetry	$\hat{D}$	0.695	0.624	0.212	0.365	1.203	0.305	12.7
	$\hat{\sigma }$	0.671	0.659	0.044	0.593	0.764	0.066	76.2
	$\lambda_{0.\text{trap}}$	0.012	0.010	0.004	0.005	0.022	0.333	41.0
SCR	$\hat{D}$	0.747	0.607	0.261	0.344	1.362	0.349	—
	$\hat{\sigma }$	0.541	0.470	0.149	0.335	0.908	0.275	—
	$\lambda_{0.\text{trap}}$	0.023	0.016	0.013	0.007	0.057	0.565	

*Note*: We present for all parameters the 95% Bayesian credible intervals (CRI); coefficient of variation (CV = SD/mean), and reduction of CV from parameters of the SCR model, and the reduction of $\lambda_{0.\mathrm{cam}}$ CV from the SCR‐occupancy model.

**FIGURE 3:**
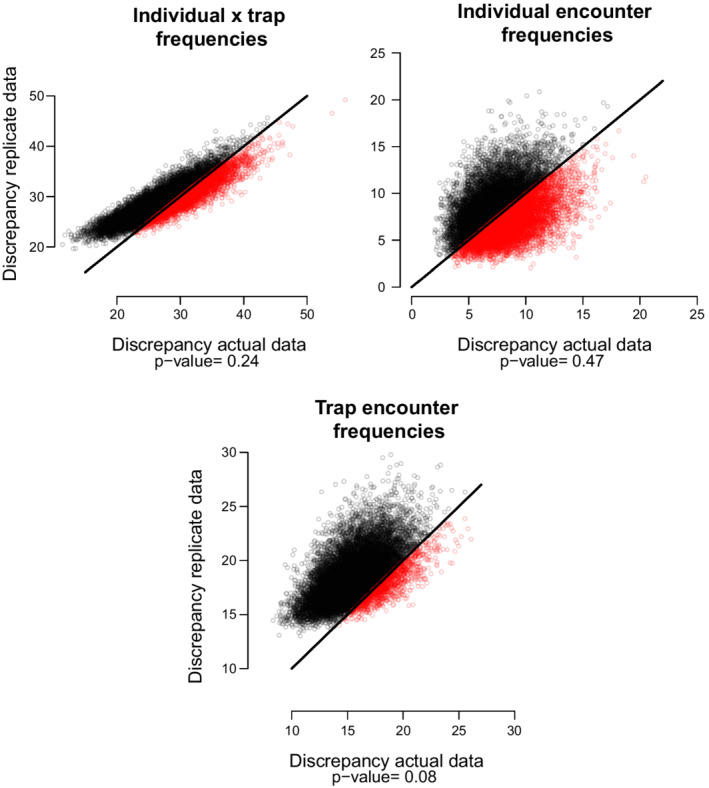
Scatter plots of discrepancy measures for the replicate versus the actual data for the spatial capture–recapture‐integrated (SCR‐IM) model. The Bayesian *p*‐values are the proportion of points below the 1:1 equality line (black). Top left: Individual × trap frequencies, which summarizes the data by individual and detector aggregated over a single occasion. Top right: Individual encounter frequencies, which assess individual heterogeneity. Bottom: Detector (trap) frequencies, which is based on aggregating over individuals and replicates to form detector‐encounter frequencies.

**FIGURE 4:**
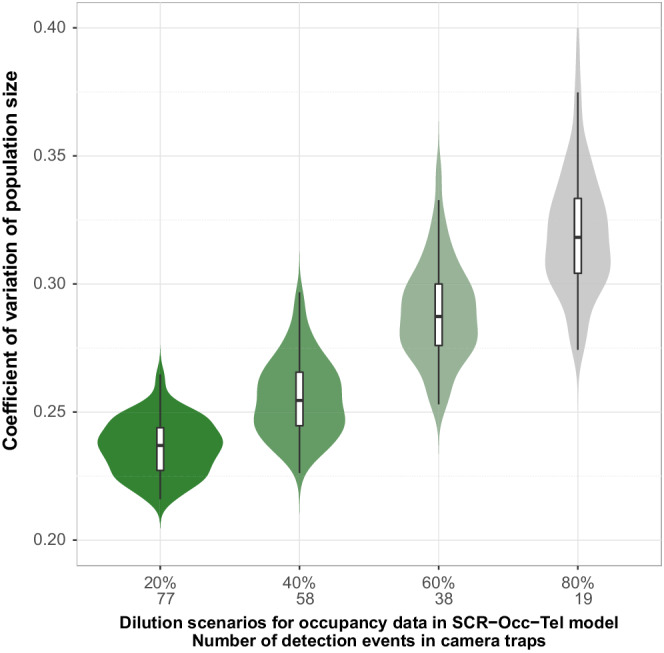
Relationships between the coefficient of variation for population size and number of detections from the occupancy submodel, under four dilution scenarios and 100 simulated datasets per scenario: 20% (77 detections), 40% (58), 60% (38), and 80% (19). Violins represent the distribution of CVs in each dilution scenario.

The GoF test did not detect lack of model fit for the SCR‐Occ‐Tel, as the BPVs are not close to 0 or 1 for the first and second components. However, we detected a relative lack of fit in the third component of the GoF, suggesting that capture frequency per trap is not well explained by this model (Figure 3). We found sample size in the occupancy model to be negatively correlated with the coefficient of variation for density estimates (Figure 4), indicating that a higher number of detections leads to increased precision of parameter estimates under the SCR‐Occ‐Tel approach. In our case study, ${\mathrm{CV}}_{n=19}=0.32\;\left(\mathrm{SD}:0.03\right)$ and ${\mathrm{CV}}_{n=77}=0.24\;\left(\mathrm{SD}:0.01\right)$.

Our simulation study revealed better accuracy in population size and σ estimates using SCR‐Occ‐Tel when compared to the standard SCR (Table 2).

**TABLE 2:** Bias and precision of parameters estimate from (i) spatial capture–recapture, detection/nondetection, and telemetry data (SCR‐occupancy‐telemetry); (ii) spatial capture–recapture and detection/nondetection data (SCR‐occupancy); (iii) spatial capture–recapture and telemetry data (SCR telemetry); and (iv) spatial capture–recapture (SCR) data alone, in 100 simulated datasets. We used a similar of our proof‐of‐concept scenario: (i) the same box trap and camera trap deployments; (ii) similar detection numbers (97; range: 72–117); (iii) density of 0.35 individuals/km^2^; (iv) sigma of 0.6 km; (v) 4 telemetry‐tagged individuals (20 locations each); and (vi) 21 captures. We used mode as point estimate. Parameters: population density ($\hat{D}$; individuals/km^2^); spatial scale parameter of the detection function ($\hat{\sigma }$; km) and baseline detection rate for box traps (${\hat{\lambda }}_{0.\text{trap}}$).

Model	Parameter (mode)	Relative bias	RMSE
SCR‐occupancy‐telemetry	$\hat{D}$	−0.102	0.099
	$\hat{\sigma }$	−0.032	0.061
	$\lambda_{0.\text{trap}}$	−0.121	0.008
SCR‐occupancy	$\hat{D}$	−0.114	0.105
	$\hat{\sigma }$	−0.085	0.122
	$\lambda_{0.\text{trap}}$	−0.105	0.010
SCR‐telemetry	$\hat{D}$	−0.192	0.110
	$\hat{\sigma }$	−0.025	0.060
	$\lambda_{0.\text{trap}}$	−0.026	0.010
SCR	$\hat{D}$	−0.249	0.126
	$\hat{\sigma }$	−0.044	0.127
	$\lambda_{0.\text{trap}}$	−0.082	0.012

## 4 DISCUSSION

Our results support that, among all models fitted, the SCR‐Occ‐Tel provides the most precise parameter estimates. We demonstrate that integrating occupancy (detection/nondetection) data improve precision in the estimation of *D*, and that integrating telemetry data further contributed to increase precision in estimating the detection parameters σ, $\lambda_{0.\text{trap}}$, and $\lambda_{0.\mathrm{cam}}$. Furthermore, our simulation study also support that apart from more precise, the integrated SCR‐Occ‐Tel model also provides more accurate parameter estimates. Therefore, all combined data sources lead to a more reliable estimate of stone marten population density, even with limited sample sizes. In fact, the precision obtained for our SCR‐Occ‐Tel density estimate approaches the 20% CV threshold suggested by Pollock et al. (1990) for precise wildlife studies, and this achievement is a consequence of data integration. The improvement provided by the occupancy submodel regards to the wider state space encompassed by the camera trapping survey when compared to our box‐trap deployment. The latter might have been insufficient to capture the full extent of the marked stone martens' movement. While an integrated SCR‐Counts model could also provide auxiliary information on σ (Chandler & Royle, 2013), our reduced number of camera traps and their scattered deployment prevented the collection of detection records with required spatial correlation (Kéry & Royle, 2021, p. 633). Therefore, telemetry data were needed for a precise σ estimate, as further supported by the results of our simulation exercise (Table 2). On the other hand, we found that adding telemetry data alone to the SCR model improved the precision of the detection parameters, but its efficacy to improve D precision estimate was limited. Accordingly, if: (i) SCR detectors (box traps) are spatially deployed allowing to capture the full extent of animal movement, and (ii) the effort required to obtain enough spatial recaptures does not lead to violation of population closure assumptions, it would be preferable to design a camera‐trap sampling scheme focused in increasing occupancy data, instead of devoting an equivalent effort to telemetry tagging a small subset of animals. A distance of 1.5σ−2σ (970–1300 m in our study for stone marten) between box traps (Efford, 2004; Sollmann et al., 2012; Sun et al., 2014) would have improved the sampling design to cover the movement of the target species, and perhaps then, integration of telemetry data might have been unnecessary.

In general, the results of the simulations are consistent with those of the proof‐of‐concept approach. The models fitted to our simulated datasets also show better accuracy of the SCR‐Occ‐Tel model in estimating population size when compared to the standard SCR model. Moreover, if we compare the relative contributions of the occupancy and the telemetry submodels, the improvement is similar in terms to the RMSE reduction, but the former provides higher bias reduction. As expected, the telemetry submodel led to an important RMSE improvement in sigma estimates, which is particularly evident in the SCR‐Tel model. The larger $\lambda_{0.\text{trap}}$ bias in SCR‐Occ‐Tel is probably due to the additional information in SCR‐Occ coming from telemetry values—that induces higher and more realistic sigma values—since in SCR models σ and $\lambda_{0}$ are structurally correlated, and changes in former parameter are compensated by reciprocal variation in the magnitude of detection (Efford & Mowat, 2014).

As expected, we found the baseline detection rate in cameras ($\lambda_{0.\mathrm{cam}}$) to be higher than with box traps ($\lambda_{0.\text{trap}}$), which has important implications for planning further studies targeting stone martens. The baseline detection rate with box traps was half the baseline detection rate with camera traps (0.014; SD: 0.004). The relatively high detection rate via camera traps (0.027; SD: 0.007) is encouraging, as it suggests that obtaining suitable occupancy dataset for the integrated model should be relatively easy. The use of lures is widely used to improve the detection probability of carnivores in camera‐trapping studies (e.g., Avrin et al., 2021; Holinda et al., 2020; Iannarilli et al., 2021), and lure selection can significantly affect the ability to detect rare and elusive species either by making them appearing sooner, moving closer or stay longer in the cameras field of view (Ferreras et al., 2018; Tourani et al., 2020). Therefore, an appropriate selection of the most effective lures could contribute to optimizing camera‐trapping sampling protocols (Figure 4) and lead to better precision in SCR‐IM estimates (Gerber et al., 2011; Macaulay et al., 2020).

A key assumption when integrating different datasets into a single unified modeling approach (i.e., an IM) is that the underlying state process being sampled is the same (Kéry & Royle, 2021). The remarkable overlap in occupancy estimates derived from box trapping and camera trapping do not provide grounds to suspect that different ecological processes were occurring across both spatial extents, and do not refute the “same state process” hypothesis. While we acknowledge that occupancy overlap may not suffice to ascertain the same underlying state process, we stress that the underlying processes of the data integrated in any IPM are rarely identical. However, because parameters are estimated as a weighted average based on the information from all the analyzed data sets combined (Kéry & Royle, 2021, p. 645; Schaub & Kéry, 2022, p. 239), IM's are relatively robust to discrepancies between its composing submodels (SCR, occupancy, and telemetry, in our case) and such an averaging may even be advantageous (Kéry & Royle, 2021, p. 645). We also found high overlap for all parameters estimated through the SCR, SCR‐Occ, SCR‐Tel, and SCR‐Occ‐Tel approaches, and thus none should have a disproportionate influence on the results. A point that need further studies is the integration with extra‐binomial noise or unmodeled overdispersion of the datasets (Isaac et al., 2020).

Probably owing to the challenges involved in their individual identification, stone marten density estimates are lacking in the literature. Exceptions include Balestrieri et al. (2021), who estimated 0.95 (0.7 – 1.3) individuals/km^2^ in the Alpine areas of N Italy using the R package capwire (Pennell et al., 2013). To the best of our knowledge, only two studies reported stone marten density in Mediterranean region of Iberia, both in places of Extremadura (SW Spain) and estimated $\hat{D}=0.24;\mathrm{SD}:0.08$ and $\hat{D}=0.26;\mathrm{SD}:0.14$ individuals/km^2^ for Valdecigüeñas (Jiménez et al., 2017) and Matachel Valley (Jiménez, Nuñez‐Arjona, et al., 2019), respectively. These studies used SMR (Royle et al., 2014;  Sollmann, Gardner, Parsons, et al., 2013) and gen‐SMR (Whittington et al., 2017) models with integrated telemetry data, respectively, and consequently allowed estimating movement parameters $\hat{\sigma }=0.82;\mathrm{SD}:0.05$ (Jiménez et al., 2017) and $\hat{\sigma }=0.67;\mathrm{SD}:0.06$ km (Jiménez, Nuñez‐Arjona, et al., 2019). Our estimate is the most precise among those published so far for stone martens in Mediterranean areas and conforms with expectations based on the conditions of the landscape. Density estimates in Cabañeros National Park, in the Mesomediterranean subhumid area (Rivas‐Martínez, 1983) with extensive areas of strawberry trees, would be consistent with previously obtained densities in Mesomediterranean xeric areas (Valdecigüeñas and Matachel Valley) both with a lower production of fleshy fruited plants. The ecological importance of the stone marten in Mediterranean environments is largely unknown, but it is assumed to play a key role as a fleshy fruit disperser (Pereira et al., 2019), potentially acting as an ecosystem engineer (González‐Varo et al., 2013). Therefore, accurately estimating its density and the factors that govern it, such as the habitat and food availability, and intra‐specific relationships (Jiménez, Nuñez‐Arjona, et al., 2019; Monterroso et al., 2020), are paramount from an ecological standpoint due to the multitrophic relationships and implications for ecosystem structure. Although density estimation of stone martens would be possible using SMR (incorporating information from box‐trap captures into the gen‐SMR model), the challenges involved in marking stone martens with reasonable mark permanence and easy recognition (e.g., using collars or ear tags) make the use of transponders more appropriate. However, maintaining a large enough box‐trapping sampling grid targeting an SCR approach is laborious, expensive, and invasive for all captured individuals. Under these conditions, the use of the SCR‐Occ‐Tel is recommended because it allows working with a relatively small capture–recapture grid and handling smaller numbers of animals, while complementing it with detection (cheaper) data collected over a larger area. As recaptures are based on transponder reading, a possible methodological improvement would be the deployment of a network of automatic transponder readers (Charney et al., 2009) as a new set of detectors, which would collect data to complement live captures in box traps and presence/absence in camera traps. This would require, however, including a new parameter in the model for baseline detection rate at transponder readers.

With this study, we provide a proof of concept of the SCR‐Occ‐Tel model with real data, and of its applicability to an elusive species with near‐absent natural markings by integrating multiple data sources with small sample size. Our results indicate that the SCR‐Occ‐Tel model could be used for other species in which individual recognition in camera traps is not possible, minimizing invasive sampling procedures. Possible extensions to this approach, for example, by combining occupancy and SCR with categorical covariates (Augustine et al., 2019) or random‐thinning SCR (Jiménez et al., 2021), may allow additional improvements in the precision of the estimates. The integration possibilities are as many as the possible problems to be addressed, and we are only at the beginning of this journey.

## AUTHOR CONTRIBUTIONS

**José Jiménez:** Conceptualization (lead); formal analysis (lead); funding acquisition (equal); methodology (lead); software (lead); writing – original draft (lead). **Francisco Díaz‐Ruiz:** Data curation (equal); investigation (equal); writing – review and editing (equal). **Pedro Monterroso:** Data curation (equal); investigation (equal); writing – review and editing (equal). **Jorge Tobajas:** Data curation (equal); investigation (equal); writing – review and editing (equal). **Pablo Ferreras:** Data curation (equal); funding acquisition (lead); investigation (equal); project administration (lead); supervision (lead); writing – original draft (supporting); writing – review and editing (equal).

## CONFLICT OF INTEREST

The authors declared no potential conflicts of interest with respect to the research, authorship, and/or publication of this article.

## Supporting information


Appendix S1

## Data Availability

R script and dataset analyzed during the current study are available in the Zenodo repository at: https://doi.org/10.5281/zenodo.6976873.
