# Supplementary material for: Occupancy data improves parameter precision in spatial capture–recapture models
Source: Ecol Evol. 2022 Aug 26;12(8):e9250. doi: 10.1002/ece3.9250 (PMC9412271; doi:10.1002/ece3.9250)
Supplement: Supplementary file 1 — Appendix S1 [file ECE3-12-e9250-s001.docx]

Improving density estimate in spatial capture-recapture procedures with occupancy data

Appendix

April, 20 2022

#### José Jiménez${}^{1}$, Francisco Díaz-Ruiz${}^{2}$, Pedro Monterroso${}^{3,4}$, Jorge Tobajas${}^{1}$, Pablo Ferreras${}^{1}$

${}^{1}$ Instituto de Investigación en Recursos Cinegéticos (IREC, CSIC-UCLM-JCCM), Ronda de Toledo 12, 13071 Ciudad Real, Spain.

${}^{2}$ Departamento de Biología Animal, Facultad de Ciencias, Universidad de Málaga, 29071 Málaga, Spain.

${}^{3}$ CIBIO, Centro de Investigacão em Biodiversidade e Recursos Genéticos, InBIO Laboratório Associado, Campus de Vairão, Universidade do Porto, 4485-661 Vairão, Portugal.

^4^ BIOPOLIS Program in Genomics, Biodiversity and Land Planning, CIBIO, Campus de Vairão, 4485-661 Vairão, Portugal.

# 1. BUGS Codes

## 1. SCR-Integrated Model (full model)

library(nimble)
## define the model
code <- nimbleCode({

 p0.trp ~ dunif(0,1)
 p0.cam ~ dunif(0,5)
 sigma ~ dunif(0,100)
 psi ~ dunif(0,1)

 # Capture histories and euclidean distances
 for(i in 1:M){
 z[i] ~ dbern(psi)
 s[i,1] ~ dunif(xlim[1],xlim[2])
 s[i,2] ~ dunif(ylim[1],ylim[2])
 dtrap[i,1:J.trap] <- (s[i,1]-X.trap[1:J.trap,1])^2 + (s[i,2]-X.trap[1:J.trap,2])^2
 p[i,1:J.trap] <-p0.trp* exp(- dtrap[i,1:J.trap]/(2*sigma^2))
 dcam[i,1:J.cam] <- (s[i,1] - X.cam[1:J.cam,1])^2 + (s[i,2] - X.cam[1:J.cam,2])^2
 lam[i,1:J.cam] <- p0.cam*exp(-dcam[i,1:J.cam] /(2*sigma^2))

 # Sub-model for spatial capture-recapture
 for(j in 1:J.trap){
 pscr[i,j] <- 1 - exp(-p[i,j])
 for(k in 1:K.trap){
 mu[i,j,k]<- pscr[i,j]*Oper.trap[j,k]*z[i]
 y[i,j,k] ~ dbern(mu[i,j,k])
 }
 }
 # Compute detection probability for occupancy
 for(j in 1:J.cam) {
 pocc[i,j] <- 1 - exp(-lam[i,j])
 # for occupancy data compute probability of not captured
 pn[i,j] <- (1 - (pocc[i,j]*z[i]) )
 } #j
 }
 # Sub-model for the detection-nondetection data
 for(j in 1:J.cam) {
 for(k in 1:K.cam){
 mu2[j,k]<-(1-prod(pn[1:M,j]))*Oper.cam[j,k]
 yocc[j,k] ~ dbern(mu2[j,k])
 }#k
 }#j

 # Sub-model for telemetry
 for (r in 1:nlocs){
 locs[r,1]~dnorm(s[inds[r],1], 1/(sigma^2))
 locs[r,2]~dnorm(s[inds[r],2], 1/(sigma^2))
 }

 N <- sum(z[1:M])
 D <- N/area
})

## 2. Full SCR-Integrated Model (aggregated over K, with goodness-of-fit)

library(nimble)
## define the model
code <- nimbleCode({

 p0.trp ~ dunif(0,1)
 p0.cam ~ dunif(0,5)
 sigma ~ dunif(0,100)
 psi ~ dbeta(1,1)

 # Capture histories and euclidean distances
 for(i in 1:M){
 z[i] ~ dbern(psi)
 s[i,1] ~ dunif(xlim[1],xlim[2])
 s[i,2] ~ dunif(ylim[1],ylim[2])
 dtrap[i,1:J.trap] <- (s[i,1]-X.trap[1:J.trap,1])^2 + (s[i,2]-X.trap[1:J.trap,2])^2
 p[i,1:J.trap] <-p0.trp* z[i]*exp(- dtrap[i,1:J.trap]/(2*sigma^2))
 dcam[i,1:J.cam] <- (s[i,1] - X.cam[1:J.cam,1])^2 + (s[i,2] - X.cam[1:J.cam,2])^2
 lam[i,1:J.cam] <- p0.cam*exp(-dcam[i,1:J.cam]/(2*sigma^2))*z[i]

 # Sub-model for spatial capture-recapture
 for(j in 1:J.trap){
 pscr[i,j] <- 1 - exp(-p[i,j])
 y[i,j] ~ dbinom(pscr[i,j]*z[i], KT.trap[j])
 Ysim[i,j] ~ dbinom(pscr[i,j]*z[i], KT.trap[j]) # simulated
 Yexp[i,j] <-pscr[i,j]*z[i]*KT.trap[j] # expected
 # components for T1
 err1obs[i,j] <- (sqrt(y[i,j]) - sqrt(Yexp[i,j]))^2
 err1sim[i,j] <- (sqrt(Ysim[i,j]) - sqrt(Yexp[i,j]))^2
 }

 # components for T2
 err2obs[i] <- (sqrt(sum(y[i,1:J.trap])) - sqrt(sum(Yexp[i,1:J.trap])))^2
 err2sim[i] <- (sqrt(sum(Ysim[i,1:J.trap])) - sqrt(sum(Yexp[i,1:J.trap])))^2

 # Compute detection probability for occupancy
 for(j in 1:J.cam) {
 pocc[i,j] <- 1 - exp(-lam[i,j])
 # for occupancy data compute probability of not captured
 pn[i,j] <- (1 - (pocc[i,j]*z[i]) )
 } #j
 }
 # Sub-model for the detection-nondetection data
 for(j in 1:J.cam) {
 yocc[j] ~ dbinom((1-prod(pn[1:M,j])),KT.cam[j]) #
 }#j

 # Sub-model for telemetry
 for (r in 1:nlocs){
 locs[r,1]~dnorm(s[inds[r],1], 1/(sigma^2))
 locs[r,2]~dnorm(s[inds[r],2], 1/(sigma^2))
 }

 # components for T3
 for(j in 1:J.trap){
 err3obs[j] <- (sqrt(sum(y[1:M,j])) - sqrt(sum(Yexp[1:M,j])))^2
 err3sim[j] <- (sqrt(sum(Ysim[1:M,j])) - sqrt(sum(Yexp[1:M,j])))^2
 }

 # Fit diagnostics totals
 T1obs <- sum(err1obs[1:M,1:J.trap])
 T1sim <- sum(err1sim[1:M,1:J.trap])
 T2obs <- sum(err2obs[1:M])
 T2sim <- sum(err2sim[1:M])
 T3obs <- sum(err3obs[1:J.trap])
 T3sim <- sum(err3sim[1:J.trap])

 N <- sum(z[1:M])
 D <- N/area
})

## 3. Reversible Jump MCMC

library(nimble)
## define the model
code <- nimbleCode({

 sigma ~ dunif(0,100)
 psi ~ dunif(0,1)
 baseline.p ~ dunif(0,1)
 beta0<- log(baseline.p/(1-baseline.p))
 beta1 ~ dnorm(0, sd=100)
 theta ~ dunif(0,1)
 w ~ dbern(theta)
 wbeta1<- w * beta1

 for(j in 1:J.trap){
 logit(p0.trp[j]) <- beta0 + wbeta1*lure[j]
 }

 # Capture histories and euclidean distances
 for(i in 1:M){
 z[i] ~ dbern(psi)
 s[i,1] ~ dunif(xlim[1],xlim[2])
 s[i,2] ~ dunif(ylim[1],ylim[2])
 dtrap[i,1:J.trap] <- (s[i,1]-X.trap[1:J.trap,1])^2 +
 (s[i,2]-X.trap[1:J.trap,2])^2
 p[i,1:J.trap] <-p0.trp[1:J.trap]* exp(- dtrap[i,1:J.trap]/(2*sigma^2))

 for(j in 1:J.trap){
 pscr[i,j] <- 1 - exp(-p[i,j])
 y[i,j] ~ dbinom(pscr[i,j]*z[i], KT.trap[j])
 }
 }

 N <- sum(z[1:M])
 D <- N/area
})

## Data, constants and inits

load("C:/Users/Administrator/OneDrive/83 Proyecto Garduna PNC/05 R/StoneMarten.RData")

## Compilation

params<- c('beta0','beta1','psi','D','sigma')

Rmodel <- nimbleModel(code=code,
 constants=constants,
 data=data,
 inits=inits)

## Defining model

## Building model

## Setting data and initial values

## Running calculate on model
## [Note] Any error reports that follow may simply reflect missing values in model

## variables.

## Checking model sizes and dimensions

## [Note] This model is not fully initialized. This is not an error.
## To see which variables are not initialized, use model$initializeInfo().
## For more information on model initialization, see help(modelInitialization).

Rmodel$initializeInfo()

## [Note] Missing values (NAs) or non-finite values were found in model variables: baselin## e.p, beta0, theta, p0.trp, p, pscr, lifted_pscr_oBi_comma_j_cB_times_z_oBi_cB_L19.
## [Note] This is not an error, but some or all variables may need to be initialized for

## certain algorithms to operate properly.
## [Note] For more information on model initialization, see help(modelInitialization).

#Rmodel$calculate()
Cmodel <- compileNimble(Rmodel)

## Compiling
## [Note] This may take a minute.
## [Note] Use 'showCompilerOutput = TRUE' to see C++ compilation details.

conf<-configureMCMC(Rmodel, monitors = params, useConjugacy=TRUE, thin=1)

## ===== Monitors =====
## thin = 1: beta0, beta1, D, psi, sigma
## ===== Samplers =====
## RW sampler (1005)
## - sigma
## - psi
## - baseline.p
## - beta1
## - theta
## - s[] (1000 elements)
## binary sampler (501)
## - z[] (500 elements)
## - w

conf$addMonitors('w')

configureRJ(conf,
 targetNodes = c('beta1'),
 indicatorNodes =c('w'),
 control = list(mean = 0, scale = 1))

conf$removeSampler(paste("s[1:",M,", 1:2]", sep=""))
for(i in 1:M){
 conf$addSampler(target = paste("s[",i,", 1:2]", sep=""),
 type = 'AF_slice',control=list(adaptive=TRUE,
 adaptScaleOnly=TRUE),
 silent = TRUE)
}

conf$removeSamplers('z')
for(node in Rmodel$expandNodeNames('z')) conf$addSampler(target = node,
 type = 'slice')


MCMC <- buildMCMC(conf)
Cmcmc <- compileNimble(MCMC, project = Rmodel)

## Compiling
## [Note] This may take a minute.
## [Note] Use 'showCompilerOutput = TRUE' to see C++ compilation details.

nb = 1000
ni = 5000 + nb
nc = 3

outNim <- runMCMC(Cmcmc, niter = ni , nburnin = nb , nchains = nc,
 setSeed = FALSE, progressBar = TRUE, inits=inits,
 samplesAsCodaMCMC = TRUE)

## Inclusion probability

samplesRJ<-as.matrix(outNim)
(posterior_inclusion_prob <- mean(samplesRJ[,'w']))

## [1] 0.02106667

## 4. Simulations

Modified from Kéry and Royle (2021) page 629-631.

library(scrbook)

rnd<-c(47,94,110,112,136,182,184,229,245,297,391,480,481,483,563,590,631,648,
 666,686,689,713,716,729,735,799,825,848,870,886,915,919,931,1032,1099,
 1121,1144,1209,1212,1240,1255,1284,1337,1344,1349,1353,1391,1431,1457,
 1494,1518,1595,1657,1851,1923,1936,1939,1963,1994,1996,2075,2132,2173,
 2210,2212,2239,2254,2322,2390,2405,2528,2529,2531,2583,2590,2628,2703,
 2749,2782,2822,2828,2865,2868,2903,2918,2926,2942,2957,2962,2980,3043,
 3060,3068,3073,3100,3121,3144,3147,3155,3221)

# Data simulation
out<- matrix(NA,nrow=100,ncol=3)
for(sim in 1:100){

 setwd('C:/Users/Administrator/OneDrive/83 Proyecto Garduna PNC/05 R')
 load("traplocs.RData")
 load("camera.RData")


 # Using the same camera-trap grid (40 camera-trap)
 head(cam)
 X.cam<- cam/1000
 X1.mean<- mean(X.cam[,1])
 X2.mean<- mean(X.cam[,2])
 X.cam[,1]<-X.cam[,1]-X1.mean
 X.cam[,2]<-X.cam[,2]-X2.mean
 (ncam<-nrow(X.cam))

 # Using the same trapping grid (60 live-traps)
 traplocs <- traplocs/1000
 traplocs[,1]<- traplocs[,1]-X1.mean
 traplocs[,2]<- traplocs[,2]-X2.mean
 (ntraps <- nrow(traplocs))

 # Define state-space of point process by buffering traps
 delta <- 2 # Buffer width
 Xl <- min(X.cam[,1] - delta) # Lower x
 Xu <- max(X.cam[,1] + delta) # Upper x
 Yl <- min(X.cam[,2] - delta) # Lower y
 Yu <- max(X.cam[,2] + delta) # Upper y

 # Distribute population of size N = 82 in the state space
 N <- 82
 K.trap <- 20 # number of days of capture
 K.cam <- 50 # number of days of camera-traps
 xlims<-c(Xl,Xu)
 ylims<-c(Yl,Yu)
 area<-diff(xlims)*diff(ylims)
 D <- N/area # 0.35 ind./km2

 # Simulate activity centers
 set.seed(rnd[sim], kind = "Mersenne-Twister") # Initialize RNGs
 sx <- runif(N, Xl, Xu) # x coordinate
 sy <- runif(N, Yl, Yu) # y coordinate
 smat <- cbind(sx, sy) # Activity center


 # Plot the state space, trap locations and individual ACs
 plot(c(Xl,Xu), c(Yl,Yu), type = 'n', xlab = "x", ylab = "y", asp = 1)
 points(smat, pch = 16, cex = 1, col="red")
 points(X.cam, pch = "+", col="blue")
 points(traplocs, pch=15, col="black")

 # Parameters for the SCR model simulation
 p0.trp <- 0.03 # Baseline encounter rate
 p0.cam <- 0.25
 sigma <- 0.6 # Half-normal detection scale

 # Generate the encounters of every individual in every live-trap
 D1 <- e2dist(smat, traplocs) # Ind/trap distance matrix
 muy1 <- p0.trp * exp(-(D1*D1) / (2 * sigma^2))
 Y1 <- matrix(NA, nrow = N, ncol = ntraps)
 for(i in 1:N){
 Y1[i,] <- rpois(ntraps, K.trap*muy1[i,])
 }
 # Now take the SCR data from the inner traps
 Yscr <- Y1
 # Captured individuals appear in the data set
 totalcaps <- apply(Yscr, 1, sum)
 Yscr <- Yscr[totalcaps > 0,]
 nind<-dim(Yscr)[1]
 sum(Yscr)

 # Generate the encounters of every individual in every camera-trap
 D2 <- e2dist(smat, X.cam) # Ind/trap distance matrix
 muy2 <- p0.cam * exp(-(D2*D2) / (2 * sigma^2))
 Y2 <- matrix(NA, nrow = N, ncol = ncam)
 for(i in 1:N){
 Y2[i,] <- rpois(ncam, K.cam*muy2[i,])
 }

 # Only detections (i.e., 'degraded') data appear in the other data set
 Yocc <- Y2
 # Total number of detections observed in outer cams
 n <- apply(Yocc, 2, sum)
 X.trap<-traplocs; J.trap<-nrow(X.trap)
 X.cam <-X.cam ; J.cam <-nrow(X.cam)

 # Set up data augmentation for the encounter histories
 M <- 500
 Yaug <- array(0, dim = c(M, J.trap))
 Yaug[1:nind,] <- Yscr

 # Now convert to binary presence/absence (or occupancy) data:
 Yocc[Yocc > 1] <- 1
 sum(Yocc)

 # Telemetry data
 n.collar <- 4
 numlocs <- 20
 locs.array <- array(NA, dim=c(n.collar, 80, 2))
 for (i in 1:n.collar) {
 locs.array[i, , 1] <- rnorm(numlocs, smat[i, 1], sigma)
 locs.array[i, , 2] <- rnorm(numlocs, smat[i, 2], sigma)
 }

 for(i in 1:4){
 points(locs.array[i,,],col=i, pch=16,cex=0.85)
 }

 locs<-rbind(locs.array[1,,],locs.array[2,,],locs.array[3,,],locs.array[4,,])
 inds<-c(rep(1,20),rep(2,20),rep(3,20),rep(4,20))

## SCR approach
 ##===============
 library(nimble)
 ## define the model
 code <- nimbleCode({

 p0.trp ~ dunif(0,1)
 sigma ~ dunif(0,100)
 psi ~ dunif(0,1)

 # Capture historials and euclidean distances
 for(i in 1:M){
 z[i] ~ dbern(psi)
 s[i,1] ~ dunif(xlim[1],xlim[2])
 s[i,2] ~ dunif(ylim[1],ylim[2])

 for(j in 1:J.trap){
 dtrap[i,j] <- (s[i,1]-X.trap[j,1])^2 + (s[i,2]-X.trap[j,2])^2
 p[i,j] <- p0.trp* exp(-dtrap[i,j]/(2*sigma^2))
 pscr[i,j] <- 1 - exp(-p[i,j])
 yscr[i,j] ~ dbinom(pscr[i,j]*z[i], K.trap)
 }
 }

 N <- sum(z[1:M])
 D <- N/area
 })


 str(constants <- list(M = M,
 J.trap=J.trap,
 area=area,
 K.trap=20,
 xlim=xlims,
 ylim=ylims))

 str ( data <- list (yscr = Yaug,
 X.trap=X.trap))


 ## Init for s
 sst <- cbind(runif(M,xlims[1],xlims[2]),runif(M,ylims[1],ylims[2]))
 for(i in 1:nind){
 sst[i,1] <- mean( X.trap[Yscr[i,]>0,1] )
 sst[i,2] <- mean( X.trap[Yscr[i,]>0,2] )
 }
 sstm<-cbind(runif(4,xlims[1],xlims[2]),runif(4,ylims[1],ylims[2]))
 zst<-rep(1,M)

 str ( inits <- list (p0.trp=0.10,
 sigma=0.5,
 psi=0.3,
 s=sst,
 z=zst))

 params<- c('p0.trp','psi','N','D','sigma')

 Rmodel <- nimbleModel(code=code,
 constants=constants,
 data=data,
 inits=inits)
 Rmodel$initializeInfo()
 #Rmodel$calculate()
 Cmodel <- compileNimble(Rmodel)
 conf<-configureMCMC(Rmodel, monitors = params, useConjugacy=TRUE, thin=1)
 conf$removeSampler(paste("s[1:",M,", 1:2]", sep=""))
 for(i in 1:M){
 conf$addSampler(target = paste("s[",i,", 1:2]", sep=""),
 type = 'AF_slice',control=list(adaptive=TRUE,
 adaptScaleOnly=TRUE),
 silent = TRUE)
 }
 conf$removeSamplers('z')
 for(node in Rmodel$expandNodeNames('z')) conf$addSampler(target = node,
 type = 'slice')

 MCMC <- buildMCMC(conf)
 Cmcmc <- compileNimble(MCMC, project = Rmodel)

 nb = 1000
 ni = 5000 + nb
 nc = 3

 outNim <- runMCMC(Cmcmc, niter = ni , nburnin = nb , nchains = nc,
 setSeed = FALSE, progressBar = TRUE,
 samplesAsCodaMCMC = TRUE)

 save(outNim, file=paste("out",sim,"NimSCR.RData", sep=""))


 ## SCR-Occ approach
 ##=================
 library(nimble)
 ## define the model
 code <- nimbleCode({

 p0.trp ~ dunif(0,1)
 p0.cam ~ dunif(0,100)
 sigma ~ dunif(0,5)
 psi ~ dunif(0,1)

 # Capture historials and euclidean distances
 for(i in 1:M){
 z[i] ~ dbern(psi)
 s[i,1] ~ dunif(xlim[1],xlim[2])
 s[i,2] ~ dunif(ylim[1],ylim[2])

 for(j in 1:J.trap){
 dtrap[i,j] <- (s[i,1]-X.trap[j,1])^2 + (s[i,2]-X.trap[j,2])^2
 p[i,j] <- p0.trp* exp(-dtrap[i,j]/(2*sigma^2))
 pscr[i,j] <- 1 - exp(-p[i,j])
 yscr[i,j] ~ dbinom(pscr[i,j]*z[i], K.trap)
 }
 # Compute detection probability for occupancy
 for(j in 1:J.cam) {
 dcam[i,j] <- (s[i,1]-X.cam[j,1])^2 + (s[i,2]-X.cam[j,2])^2
 lam[i,j] <- p0.cam*exp(-dcam[i,j]/(2*sigma^2))
 pocc[i,j] <- 1 - exp(-lam[i,j])
 # for PA data compute probability of not captured
 pn[i,j] <- (1 - (pocc[i,j]*z[i]) )
 } #j
 }
 # Model for the presence-absence data
 for(j in 1:J.cam) {
 yocc[j] ~ dbinom((1-prod(pn[1:M,j])),K.cam) #
 }#j

 N <- sum(z[1:M])
 D <- N/area
 })

 str(constants <- list(M = M,
 J.trap=J.trap,
 J.cam=J.cam,
 area=area,
 K.trap=20,
 K.cam =50,
 xlim=xlims,
 ylim=ylims))

 str ( data <- list (yscr = Yaug,
 yocc=apply(Yocc,1,sum),
 X.trap=X.trap,
 X.cam=X.cam))

 ## Init for s
 sst <- cbind(runif(M,xlims[1],xlims[2]),runif(M,ylims[1],ylims[2]))
 for(i in 1:nind){
 sst[i,1] <- mean( X.trap[Yscr[i,]>0,1] )
 sst[i,2] <- mean( X.trap[Yscr[i,]>0,2] )
 }
 zst<-rep(1,M)

 str ( inits <- list (p0.trp=0.10,
 p0.cam=0.05,
 sigma=0.5,
 psi=0.3,
 s=sst,
 z=zst))

 params<- c('p0.trp','p0.cam','psi','N','D','sigma')

 Rmodel <- nimbleModel(code=code,
 constants=constants,
 data=data,
 inits=inits)
 Rmodel$initializeInfo()
 Rmodel$calculate()
 Cmodel <- compileNimble(Rmodel)

 conf<-configureMCMC(Rmodel, monitors = params, useConjugacy=TRUE, thin=1)
 conf$removeSampler(paste("s[1:",M,", 1:2]", sep=""))
 for(i in 1:M){
 conf$addSampler(target = paste("s[",i,", 1:2]", sep=""),
 type = 'AF_slice',control=list(adaptive=TRUE,
 adaptScaleOnly=TRUE),
 silent = TRUE)
 }
 conf$removeSamplers('z')
 for(node in Rmodel$expandNodeNames('z')) conf$addSampler(target = node,
 type = 'slice')
 MCMC <- buildMCMC(conf)
 Cmcmc <- compileNimble(MCMC, project = Rmodel)

 nb = 1000
 ni = 5000 + nb
 nc = 3

 outNim <- runMCMC(Cmcmc, niter = ni , nburnin = nb , nchains = nc,
 setSeed = FALSE, progressBar = TRUE,
 samplesAsCodaMCMC = TRUE)

 save(outNim, file=paste("out",sim,"NimSCR-Occ.RData", sep=""))

## SCR-Tel approach
 ## ==================
 library(nimble)
 ## define the model
 code <- nimbleCode({

 p0.trp ~ dunif(0,1)
 p0.cam ~ dunif(0,100)
 sigma ~ dunif(0,5)
 psi ~ dunif(0,1)

 # Capture historials and euclidean distances
 for(i in 1:M){
 z[i] ~ dbern(psi)
 s[i,1] ~ dunif(xlim[1],xlim[2])
 s[i,2] ~ dunif(ylim[1],ylim[2])

 for(j in 1:J.trap){
 dtrap[i,j] <- (s[i,1]-X.trap[j,1])^2 + (s[i,2]-X.trap[j,2])^2
 p[i,j] <- p0.trp* exp(-dtrap[i,j]/(2*sigma^2))
 pscr[i,j] <- 1 - exp(-p[i,j])
 yscr[i,j] ~ dbinom(pscr[i,j]*z[i], K.trap)
 }
 }

 for(t in 1:4){
 sm[t,1] ~ dunif(xlim[1],xlim[2])
 sm[t,2] ~ dunif(ylim[1],ylim[2])
 }

 # Telemetry data for n.collar animals
 for (r in 1:nlocs){
 locs[r,1]~dnorm(sm[inds[r],1], 1/(sigma^2))
 locs[r,2]~dnorm(sm[inds[r],2], 1/(sigma^2))
 }

 N <- sum(z[1:M])
 D <- N/area
 })

 str(constants <- list(M = M,
 J.trap=J.trap,
 area=area,
 K.trap=20,
 xlim=xlims,
 ylim=ylims,
 nlocs=80,
 inds=inds))

 str ( data <- list (yscr = Yaug,
 X.trap=X.trap,
 locs=locs))

 ## Init for s
 sst <- cbind(runif(M,xlims[1],xlims[2]),runif(M,ylims[1],ylims[2]))
 for(i in 1:nind){
 sst[i,1] <- mean( X.trap[Yscr[i,]>0,1] )
 sst[i,2] <- mean( X.trap[Yscr[i,]>0,2] )
 }
 sstm<-cbind(runif(4,xlims[1],xlims[2]),runif(4,ylims[1],ylims[2]))
 zst<-rep(1,M)

 str ( inits <- list (p0.trp=0.10,
 sigma=0.5,
 psi=0.3,
 s=sst,
 sm=sstm,
 z=zst))

 params<- c('p0.trp','p0.cam','psi','N','D','sigma')

 Rmodel <- nimbleModel(code=code,
 constants=constants,
 data=data,
 inits=inits)
 Rmodel$initializeInfo()
 Rmodel$calculate()
 Cmodel <- compileNimble(Rmodel)

 conf<-configureMCMC(Rmodel, monitors = params, useConjugacy=TRUE, thin=1)
 conf$removeSampler(paste("s[1:",M,", 1:2]", sep=""))
 for(i in 1:M){
 conf$addSampler(target = paste("s[",i,", 1:2]", sep=""),
 type = 'AF_slice',control=list(adaptive=TRUE,
 adaptScaleOnly=TRUE),
 silent = TRUE)
 }
 conf$removeSamplers('z')
 for(node in Rmodel$expandNodeNames('z')) conf$addSampler(target = node,
 type = 'slice')
 MCMC <- buildMCMC(conf)
 Cmcmc <- compileNimble(MCMC, project = Rmodel)

 nb = 1000
 ni = 5000 + nb
 nc = 3

 outNim <- runMCMC(Cmcmc, niter = ni , nburnin = nb , nchains = nc,
 setSeed = FALSE, progressBar = TRUE,
 samplesAsCodaMCMC = TRUE)

 save(outNim, file=paste("out",sim,"NimSCR_Tel.RData", sep=""))


 ## SCR-Occ-Tel approach
 ##=======================
 library(nimble)
 ## define the model
 code <- nimbleCode({

 p0.trp ~ dunif(0,1)
 p0.cam ~ dunif(0,100)
 sigma ~ dunif(0,5)
 psi ~ dunif(0,1)

 # Capture historials and euclidean distances
 for(i in 1:M){
 z[i] ~ dbern(psi)
 s[i,1] ~ dunif(xlim[1],xlim[2])
 s[i,2] ~ dunif(ylim[1],ylim[2])

 for(j in 1:J.trap){
 dtrap[i,j] <- (s[i,1]-X.trap[j,1])^2 + (s[i,2]-X.trap[j,2])^2
 p[i,j] <- p0.trp* exp(-dtrap[i,j]/(2*sigma^2))
 pscr[i,j] <- 1 - exp(-p[i,j])
 yscr[i,j] ~ dbinom(pscr[i,j]*z[i], K.trap)
 }
 # Compute detection probability for occupancy
 for(j in 1:J.cam) {
 dcam[i,j] <- (s[i,1]-X.cam[j,1])^2 + (s[i,2]-X.cam[j,2])^2
 lam[i,j] <- p0.cam*exp(-dcam[i,j]/(2*sigma^2))
 pocc[i,j] <- 1 - exp(-lam[i,j])
 # for PA data compute probability of not captured
 pn[i,j] <- (1 - (pocc[i,j]*z[i]) )
 } #j
 }
 # Model for the presence-absence data
 for(j in 1:J.cam) {
 yocc[j] ~ dbinom((1-prod(pn[1:M,j])),K.cam) #
 }#j

 for(t in 1:4){
 sm[t,1] ~ dunif(xlim[1],xlim[2])
 sm[t,2] ~ dunif(ylim[1],ylim[2])
 }

 # Telemetry data for n.collar animals
 for (r in 1:nlocs){
 locs[r,1]~dnorm(sm[inds[r],1], 1/(sigma^2))
 locs[r,2]~dnorm(sm[inds[r],2], 1/(sigma^2))
 }

 N <- sum(z[1:M])
 D <- N/area
 })

 str(constants <- list(M = M,
 J.trap=J.trap,
 J.cam=J.cam,
 area=area,
 K.trap=20,
 K.cam =50,
 xlim=xlims,
 ylim=ylims,
 nlocs=80,
 inds=inds))

 str ( data <- list (yscr = Yaug,
 yocc=apply(Yocc,1,sum),
 X.trap=X.trap,
 X.cam=X.cam,
 locs=locs))

 ## Init for s
 sst <- cbind(runif(M,xlims[1],xlims[2]),runif(M,ylims[1],ylims[2]))
 for(i in 1:nind){
 sst[i,1] <- mean( X.trap[Yscr[i,]>0,1] )
 sst[i,2] <- mean( X.trap[Yscr[i,]>0,2] )
 }
 sstm<-cbind(runif(4,xlims[1],xlims[2]),runif(4,ylims[1],ylims[2]))
 zst<-rep(1,M)

 str ( inits <- list (p0.trp=0.10,
 p0.cam=0.05,
 sigma=0.5,
 psi=0.3,
 s=sst,
 sm=sstm,
 z=zst))

 params<- c('p0.trp','p0.cam','psi','N','D','sigma')

 Rmodel <- nimbleModel(code=code,
 constants=constants,
 data=data,
 inits=inits)
 Rmodel$initializeInfo()
 Rmodel$calculate()
 Cmodel <- compileNimble(Rmodel)

 conf<-configureMCMC(Rmodel, monitors = params, useConjugacy=TRUE, thin=1)
 conf$removeSampler(paste("s[1:",M,", 1:2]", sep=""))
 for(i in 1:M){
 conf$addSampler(target = paste("s[",i,", 1:2]", sep=""),
 type = 'AF_slice',control=list(adaptive=TRUE,
 adaptScaleOnly=TRUE),
 silent = TRUE)
 }
 conf$removeSamplers('z')
 for(node in Rmodel$expandNodeNames('z')) conf$addSampler(target = node,
 type = 'slice')
 MCMC <- buildMCMC(conf)
 Cmcmc <- compileNimble(MCMC, project = Rmodel)

 nb = 1000
 ni = 5000 + nb
 nc = 3

 outNim <- runMCMC(Cmcmc, niter = ni , nburnin = nb , nchains = nc,
 setSeed = FALSE, progressBar = TRUE,
 samplesAsCodaMCMC = TRUE)

 save(outNim, file=paste("out",sim,"NimSCR-Occ-Tel.RData", sep=""))
}

##
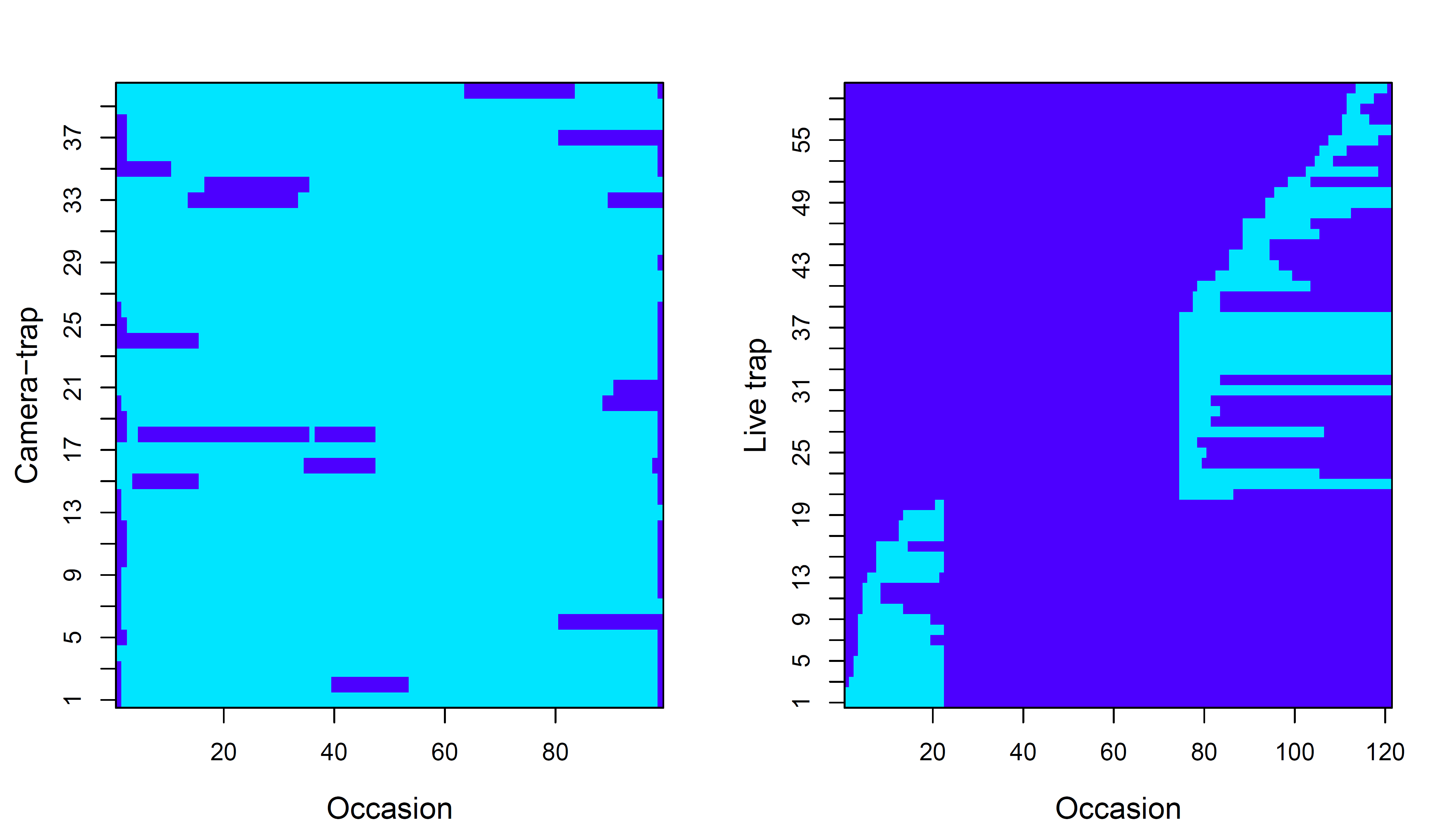


**Figure S1.** Camera-traps (left) and box-trap (right) operation plot. Light and dark blue are active and inactive detectors, respectively.

**Figure S2.** Stone marten captures (grey) and detections (golden) in box-traps (black triangles) and camera-traps (blue crosses), respectively. The sizes of the circles represent the number of captures and detections at each detector. Red points are average of captures locations for each individual, and black segment, extreme capture locations (coordinates scaled and centered).

**Figure S3.** Overlap of density probabilities for occupancy ($\psi$) using the box-traps (in grey) and camera-traps (in golden).
